# Hyperoxia Exacerbates Postnatal Inflammation-Induced Lung Injury in Neonatal BRP-39 Null Mutant Mice Promoting the M1 Macrophage Phenotype

**DOI:** 10.1155/2013/457189

**Published:** 2013-11-17

**Authors:** Mansoor A. Syed, Vineet Bhandari

**Affiliations:** Division of Perinatal Medicine, Department of Pediatrics, Yale University School of Medicine, 333 Cedar Street, New Haven, CT 06520-8064, USA

## Abstract

*Rationale*. Hyperoxia exposure to developing lungs—critical in the pathogenesis of bronchopulmonary dysplasia—may augment lung inflammation by inhibiting anti-inflammatory mediators in alveolar macrophages. *Objective*. We sought to determine the O_2_-induced effects on the polarization of macrophages and the role of anti-inflammatory BRP-39 in macrophage phenotype and neonatal lung injury. *Methods*. We used RAW264.7, peritoneal, and bone marrow derived macrophages for polarization (M1/M2) studies. For *in vivo* studies, wild-type (WT) and BRP-39^−/−^ mice received continuous exposure to 21% O_2_ (control mice) or 100% O_2_ from postnatal (PN) 1 to PN7 days, along with intranasal lipopolysaccharide (LPS) administered on alternate days (PN2, -4, and -6). Lung histology, bronchoalveolar lavage (BAL) cell counts, BAL protein, and cytokines measurements were performed. *Measurements and Main Results*. Hyperoxia differentially contributed to macrophage polarization by enhancing LPS induced M1 and inhibiting interleukin-4 induced M2 phenotype. BRP-39 absence led to further enhancement of the hyperoxia and LPS induced M1 phenotype. In addition, BRP-39^−/−^ mice were significantly more sensitive to LPS plus hyperoxia induced lung injury and mortality compared to WT mice. *Conclusions*. These findings collectively indicate that BRP-39 is involved in repressing the M1 proinflammatory phenotype in hyperoxia, thereby deactivating inflammatory responses in macrophages and preventing neonatal lung injury.

## 1. Introduction

Development of respiratory distress syndrome (RDS) adversely affects patient populations in neonatal intensive care units, which increases the risk of developing the chronic lung disease, bronchopulmonary dysplasia (BPD) [1]. This occurs primarily in preterm infants as a consequence of severe lung injury resulting from mechanical ventilation and oxygen exposure and is characterized by inflammation and epithelial cell death. Premature infants are also more likely to be exposed to infection; prenatal or postnatal inflammation accelerates the development of BPD by itself or combined with a variety of postnatal injuries, resulting in disruption of lung alveolar and vascular development [2–6].

Mouse breast regression protein-39 (BRP-39; Chi3l1) and its human homologue YKL-40 are chitinase-like proteins present in a variety of cells, including monocytes and macrophages, and have been shown to play a role in various macrophage mediated inflammatory diseases [7]. In our previous work, we demonstrated that the levels of tracheal YKL-40 are lower in premature babies that develop BPD or die compared with those without these complications [8].

M1 macrophage polarization is associated with inflammation and tissue destruction [9, 10], whereas the M2 macrophage has an anti-inflammatory phenotype that is associated with wound repair and angiogenesis [11, 12]. Macrophages are polarized to the M1 state by lipopolysaccharide (LPS), interferon gamma (IFN*γ*), and other stimulants which upregulate proinflammatory cytokines including interleukin- (IL-) 1**β**, IL-6, and IL-12 and increase the production of reactive oxygen species and nitrogen intermediates [13]. In contrast, macrophages are polarized to the M2 state by IL-4 [14], which upregulates scavenger receptors, mannose receptor, and IL-1 receptor antagonist [13]. M2 cells also secrete the anti-inflammatory cytokine IL-10 and downregulate the production of proinflammatory cytokines [14]. These cells also upregulate arginase-1 (Arg1), which metabolizes arginine to ornithine and polyamines, and thereby diminish the inducible nitric-oxide synthase (iNOS) reaction [9, 10].

M1 polarization supports resistance to intracellular bacteria and controls the acute phase of inflammation. However, an excessive or sustained M1 activated state is deleterious for the host, as demonstrated in acute infections and sepsis [10, 15].

Given the critical interaction of hyperoxia with inflammation in the pathogenesis of neonatal lung injury resulting in BPD, we hypothesized that hyperoxia exposure would exacerbate inflammation-induced M1 and suppress the M2 phenotype in macrophages. Our aims were to study the impact of hyperoxia exposure on LPS induced effects on the M1/M2 phenotype in *in vitro* systems. Furthermore, this study was designed to evaluate the role of macrophage polarization and BRP-39 in LPS and hyperoxia plus LPS induced injury in *in vitro* and developmentally appropriate *in vivo* lung injury models.

We show that hyperoxia in the presence of LPS promotes M1 and inhibits the M2 phenotype in macrophages. Hyperoxia and/or LPS decreases BRP-39 expression, and its deletion promotes the M1 phenotype in macrophages. Neonatal BRP-39 null mutant (BRP-39^−/−^) mice have significantly increased mortality on concomitant LPS and hyperoxia exposure. In addition, in the surviving BRP-39^−/−^ mice lungs, there are significant alveolar simplification and inflammation.

## 2. Materials and Methods

### 2.1. Cell Culture

Primary murine peritoneal macrophages, bone marrow derived macrophages (BMDMs), and the murine macrophage cell line RAW264.7 were isolated or cultured as previously described [16, 17]. LPS-EB Ultrapure (100 ng/mL; Invitrogen) and IL-4 (10 ng/mL; Cell Signaling Technology, Inc.) were used as indicated.

### 2.2. Neonatal Mice Lung Injury Model

We used C57BL6/J mice in our experimental studies. All animal work was approved by the Institutional Animal Care and Use Committee at the Yale University School of Medicine. BRP-39^−/−^ mice were generated and characterized as described earlier [8] and were a kind gift from Jack Elias, MD. Mice pups delivered on postnatal day 1 (PN1) were randomly divided into four groups: the control group, receiving saline and room air exposure, the LPS group, receiving an intranasal dose (3 **μ**g/3 **μ**L) of LPS on alternate days (PN2, -4, and -6) in room air (RA), and the LPS and hyperoxia groups, receiving an intranasal LPS dose (PN2, -4 and -6) and 100% oxygen exposure from PN1-7. After exposure to hyperoxia for 1 week, pups were killed immediately.

### 2.3. Oxygen Exposure

For the exposure to hyperoxia (100% O_2_), newborn (NB) mice (along with their mothers) were placed in cages in an airtight Plexiglas chamber (55 × 40 × 50 cm), as described previously [18–20]. Exposure to oxygen was initiated on PN1 of life. Two lactating dams were used. Mothers were alternated in hyperoxia and RA every 24 h. The litter size was kept limited to 12 pups to control for the effects of litter size on nutrition and growth. Throughout the experiment, they were given free access to food and water. Oxygen levels were constantly monitored by an oxygen sensor that was connected to a relay switch incorporated into the oxygen supply circuit. The inside of the chamber was kept at atmospheric pressure, and mice were exposed to a 12 h light-dark cycle.

### 2.4. Real-Time PCR

Total RNA was reverse-transcribed by the iScript cDNA synthesis kit (Bio-Rad), amplified using SYBR Green PCR Master Mix (Bio-Rad), and detected by the opticon 2 real-time machine (MJ Research).

### 2.5. ELISA

Supernatants and tissue homogenates were analyzed by sandwich ELISA for IL-1**β**, IL-6, and IL-10, as per manufacturer's (R&D Systems) instructions [8, 20–23].

### 2.6. Immunoblot Analysis

Detection of Arg1 (BD Biosciences), Ym1 (Chi3l3) (STEMCELL Technologies), iNOS, and **β**-actin (Santa Cruz Biotechnology Inc.) was done using appropriate antibodies by Western analysis, as described previously [18, 20, 24]. Proteins were visualized with the pico ECL Western blotting kit (Pierce), and blots were exposed to HyBlot autoradiography films (Denville Scientific Inc.).

### 2.7. Histology

Lung tissues obtained from the NB mice from the LPS and hyperoxia experiments at PN7 were subjected to a standard protocol for lung inflation and fixed overnight in 10% buffered formalin. After washing in fresh PBS, fixed tissues were dehydrated, cleared, and embedded in paraffin by routine methods. Sections (5 **μ**m) were collected on Superfrost Plus positively charged microscope slides (Fisher Scientific Co., Houston, TX, USA), deparaffinized, and stained with hematoxylin and eosin, as described previously [8, 18, 20, 25, 26].

### 2.8. Lung Morphometry

Alveolar size was estimated from the mean chord length of the airspace, as described previously [18–20]. Chord length increases with alveolar enlargement. Septal thickness was measured, as described previously [27].

### 2.9. Statistics

For the *in vitro* and animal studies, values were expressed as means ± SEM. As appropriate, groups were compared with the two-way ANOVA and corrected for multiple comparisons by the Tukey test and the logrank test (for the survival analysis), using GraphPad Prism 3.0 (GraphPad Software, Inc., San Diego, CA, USA). In all analyses, a *P* < 0.05 was considered statistically significant.

## 3. Results

### 3.1. Hyperoxia Differentially Regulates M1/M2 Phenotype in Macrophages

**Figure 1:**
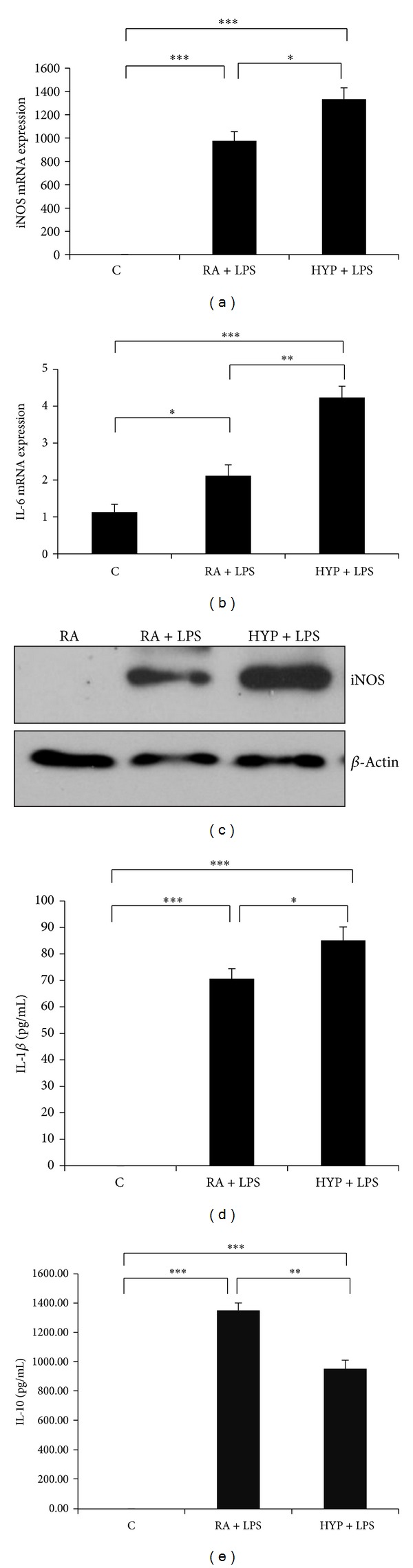
Hyperoxia promotes the M1 phenotype in macrophages. Inducible nitric oxide synthase (iNOS) and interleukin-6 (IL-6) mRNA expression were measured by real-time PCR in the RAW264.7 macrophages after stimulation with lipopolysaccharide (LPS; 100 ng/mL) for 16 h in room air (RA) or 95% hyperoxia (HYP) ((a) and (b)). Representative Western blot showing 24 h LPS mediated protein induction of iNOS in RA and HYP groups (c). IL-1*β* and IL-10 were measured by ELISA in the supernatants of macrophages after stimulation for 24 h with LPS ((d)-(e)). Results expressed as the mean ± SEM of data obtained from three independent experiments. C: control (RA). *P < 0.05, **P < 0.01, and ***P < 0.001.

To determine whether hyperoxia is critical to macrophage polarization, we first performed quantitative real-time PCR (qPCR) analysis in RAW or peritoneal macrophages after stimulation with well-established M1 (LPS) or M2 (IL-4) polarizing agents [15]. To determine whether hyperoxia affects LPS induced M1 markers, cells were stimulated with LPS in presence or absence of hyperoxia for 16 h and then iNOS and IL-6 mRNA expression were evaluated. LPS treatment led to the activation of iNOS and IL-6, as expected; however, concomitant hyperoxia augmented LPS induced iNOS and IL-6 mRNA expression (Figures 1(a) and 1(b)). These differential effects on iNOS were confirmed at the protein level by Western blot analyses (Figure 1(c)). Furthermore, hyperoxia augmented LPS induced the proinflammatory cytokine IL-1*β* and attenuated anti-inflammatory IL-10 concentrations in cell culture supernatants (Figures 1(d) and 1(e)).

**Figure 2:**
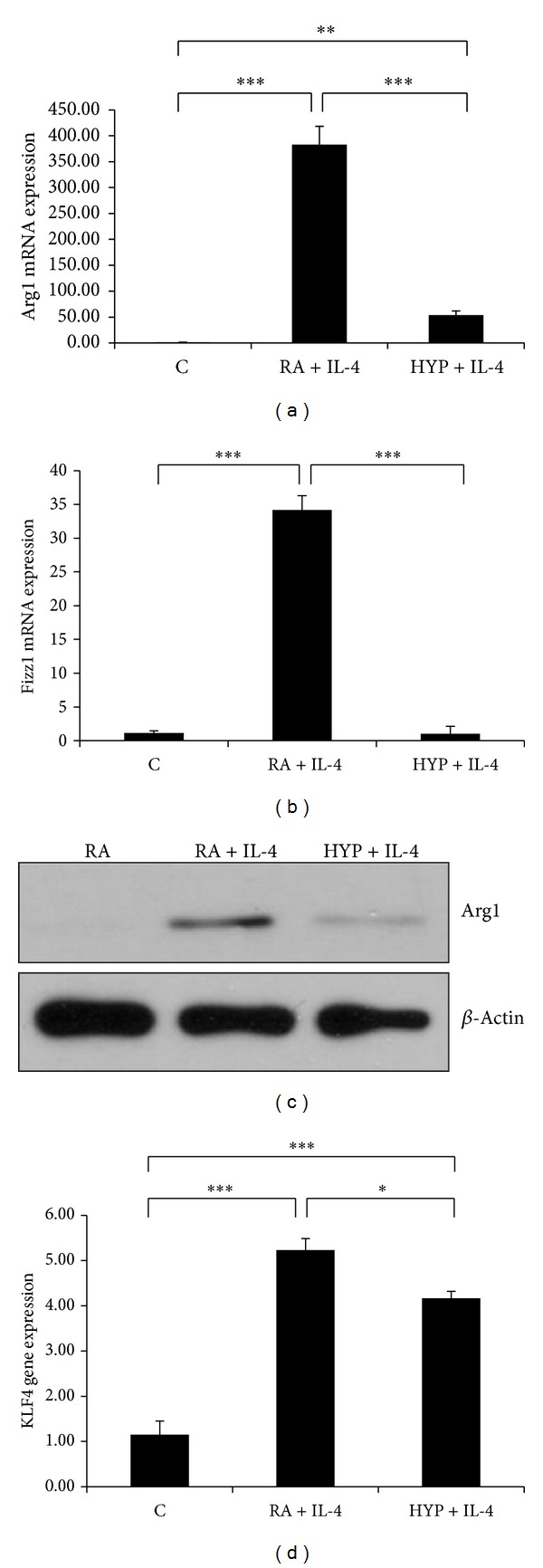
Hyperoxia inhibits the M2 phenotype in macrophages. Arg1 and Fizz1 mRNA were measured by real-time PCR in the RAW macrophages after stimulation with interleukin-4 (IL-4; 10 ng/mL) for 16 h in room air (RA) or 95% hyperoxia (HYP)((a) and (b)). Western blot showing IL-4 mediated protein induction of Arg1 in RA and HYP groups (c). KLF4 mRNA expression level was assessed by qPCR in macrophages stimulated with IL-4 in RA and HYP (d). Results expressed as the mean ± SEM of data obtained from three independent experiments. C: control (RA). *P < 0.05, ***P < 0.001.

Macrophages are highly heterogeneous cells that can quickly change their phenotype and function in response to different stimuli, and studies have documented the flexibility of macrophage activation [15]. We next investigated whether hyperoxia affects IL-4 induced M2 phenotype. Hyperoxia potently inhibited IL-4 induced M2 markers Arg1 and Fizz1 mRNA expression (Figures 2(a) and 2(b)). Western blot of Arg1 also confirmed the results of the mRNA expression (Figure 2(c)). KLF4, a novel regulator of macrophage polarization and essential for IL-4 mediated macrophage M2 phenotype [28], was also attenuated by hyperoxia (Figure 2(d)).

Taken together, our data would suggest that hyperoxia-exposure further polarizes LPS induced macrophages towards the M1 phenotype, with significant inhibition of the M2 phenotype.

### 3.2. BRP-39 Decreases with Hyperoxia and Acts as a Marker for the M2 Phenotype in Macrophages

**Figure 3:**
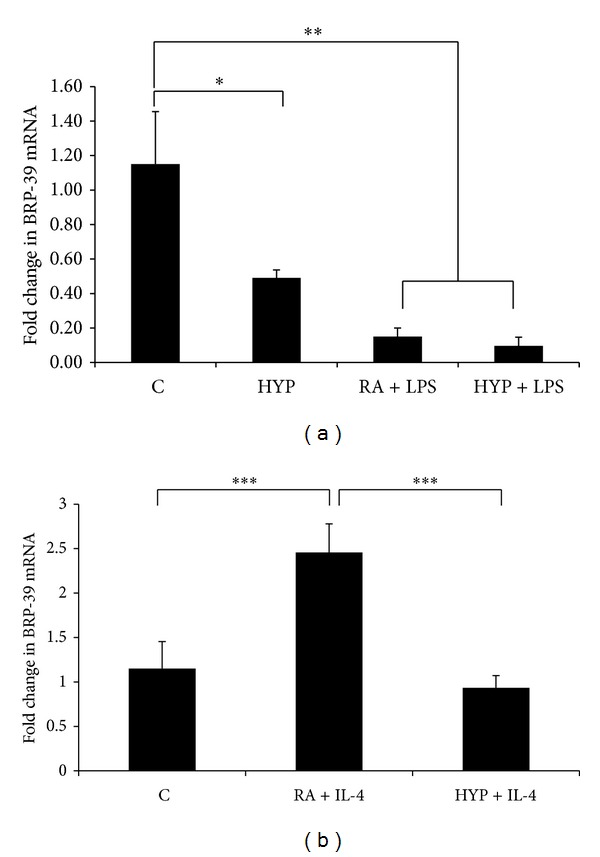
BRP-39 decreases with hyperoxia and acts as a marker for the M2 phenotype in macrophages. BRP-39 mRNA levels in mouse RAW macrophages after 16 h of stimulation with lipopolysaccharide (LPS; 100 ng/mL) (a) or interleukin-4 (IL-4; 10 ng/mL) (b). Results expressed as the mean ± SEM of data obtained from three independent experiments. C: control (RA); HYP: hyperoxia. **P < 0.01, ***P < 0.001.

Mouse breast regression protein-39 and its human homologue YKL-40 are chitinase-like proteins that have been shown to play a role in various macrophages mediated inflammatory events [29, 30]. To determine if BRP-39 gene is critical in macrophage polarization, we first performed qPCR analysis of LPS and IL-4 induced BRP-39 in RAW macrophages. These two stimuli differentially regulated the BRP-39 mRNA expression and were particularly noteworthy, as it was greatly decreased by LPS, hyperoxia, and LPS plus hyperoxia exposure (Figure 3(a)). On the other hand, BRP-39 mRNA expression was strongly enhanced by IL-4 but diminished by addition of hyperoxia (Figure 3(b)), assaying the pattern followed by other M2 phenotype markers.

### 3.3. BRP-39 Regulates M1/M2 Macrophage Polarization

**Figure 4:**
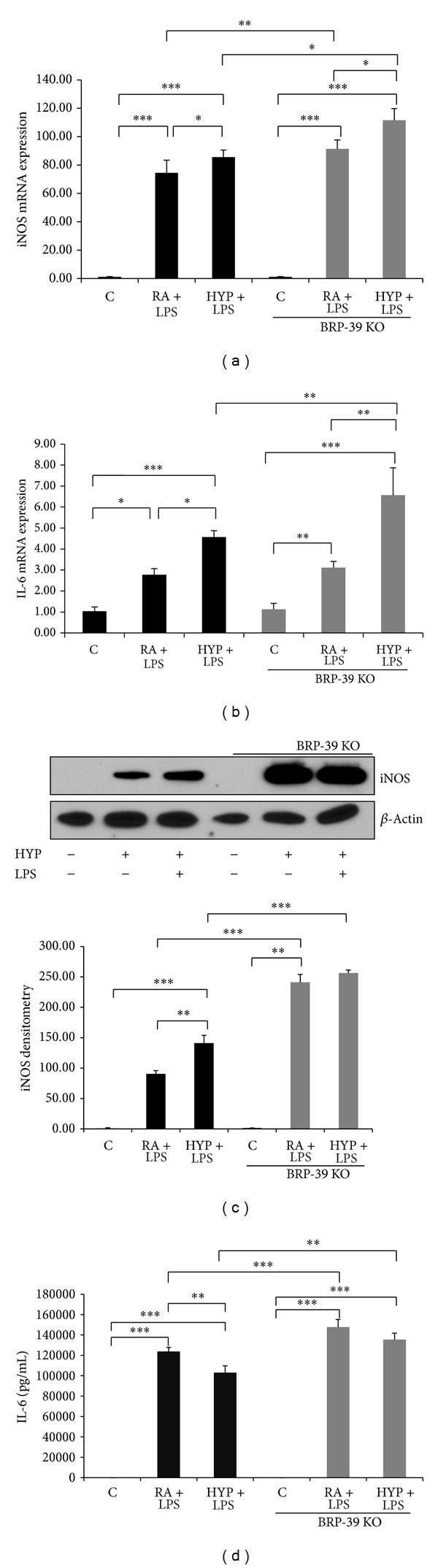
BRP-39 deletion promotes the M1 phenotype in macrophages. Enhanced expression of M1 marker genes, inducible nitric oxide synthase (iNOS), and interleukin-6 (IL-6) mRNA expression in BRP-39^−/−^ peritoneal macrophages stimulated by lipopolysaccharide (LPS; 100 ng/mL) for 16 h ((a) and (b)). Western blot and densitometry showing increased iNOS protein expression in BRP-39^−/−^ bone marrow derived macrophages (BMDMs) stimulated by LPS (100 ng/mL) or LPS plus hyperoxia for 24 h (c). BRP-39^−/−^ BMDMs generate increased IL-6 after LPS (100 ng/mL) stimulation for 16 h as compared to wild type (WT) (d). Results expressed as the mean ± SEM; *n* = 3, in each group. C: control (RA); HYP: hyperoxia; BRP39 KO: BRP39 knock out or BRP-39^−/−^. *P < 0.05, **P < 0.01, and ***P < 0.001.

To study the function of BRP-39 in the process of macrophage polarization, we utilized a loss of function approach. As noted earlier, LPS activated the M1 phenotype signaling, which was augmented by hyperoxia (Figure 1). Here, we show that upon stimulation with LPS and hyperoxia, peritoneal macrophages from BRP-39^−/−^ mice produced a higher level of iNOS and IL-6 mRNA expression than wild-type (WT) macrophages in LPS alone and LPS with hyperoxia treated groups (Figure 4(a)). These findings were further confirmed at the protein level by Western blot of iNOS (Figure 4(b)) and ELISA measurement of IL-6 (Figure 4(c)).

**Figure 5:**
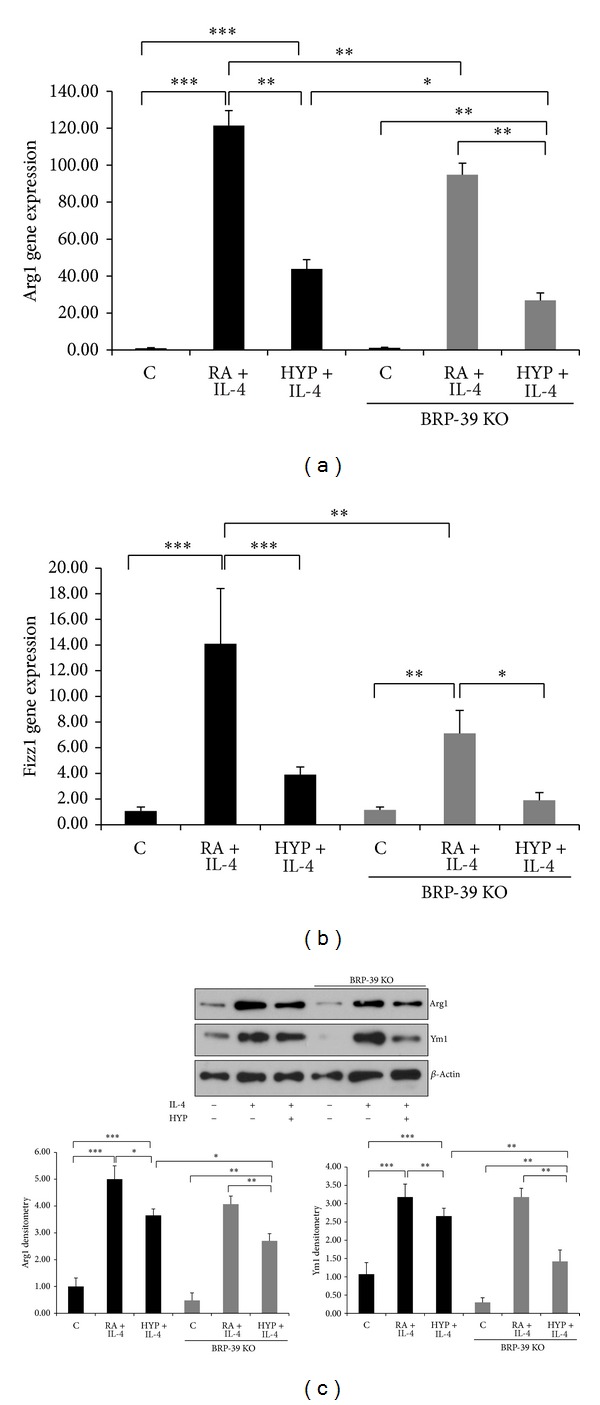
BRP-39 deletion inhibits M2 phenotype in macrophages.  Attenuated expression of M2 marker genes Arg1 and Fizz1 in BRP-39^−/−^ peritoneal macrophages stimulated with interleukin-4 (IL-4; 10 ng/mL) for 16 h ((a) and (b)). Protein levels (Western blot and densitometry) of M2 markers Arg1 and Ym1 in bone marrow derived macrophages (BMDMs) (c). Results expressed as the mean ± SEM of data obtained from three independent experiments stimulated with IL-4 (10 ng/mL) for 24 h. C: control (RA); HYP: hyperoxia; BRP39 KO: BRP39 knock out or BRP-39^−/−^. *P < 0.05, **P < 0.01, and ***P < 0.001.

To elucidate whether the differential response of BRP-39 mRNA in IL-4 and IL-4 plus hyperoxia groups (Figure 3) was due to change in macrophage polarization, we stimulated WT and BRP-39^−/−^ peritoneal macrophages and found equally robust induction of the M2 markers Arg1 and Fizz1 in the IL-4 stimulated group, which was diminished by concomitant exposure to hyperoxia in WT macrophages (Figures 5(a) and 5(b)). Similarly, IL-4 stimulated BRP-39^−/−^ macrophages also expressed high levels of Arg1 and Fizz1, which was significantly decreased compared to the WT IL-4 group (Figures 5(a) and 5(b)). However, BRP-39^−/−^ macrophages had a further significant decreased expression of Arg1 mRNA, as compared to WT, on hyperoxia plus IL-4 exposure (Figures 5(a) and 5(b)). We confirmed the results of the decrease in Arg1 expression at the protein level and noted the same pattern with the additional M2 marker, Ym1, utilizing BMDMs (Figure 5(c)).

Taken together, our data would suggest that BRP-39 is a critical regulator of the macrophage phenotype upon LPS and concomitant hyperoxia-exposure, polarizing them towards the M2 phenotype.

### 3.4. BRP-39 Protects against LPS with Concomitant Hyperoxia Induced Mortality

**Figure 6:**
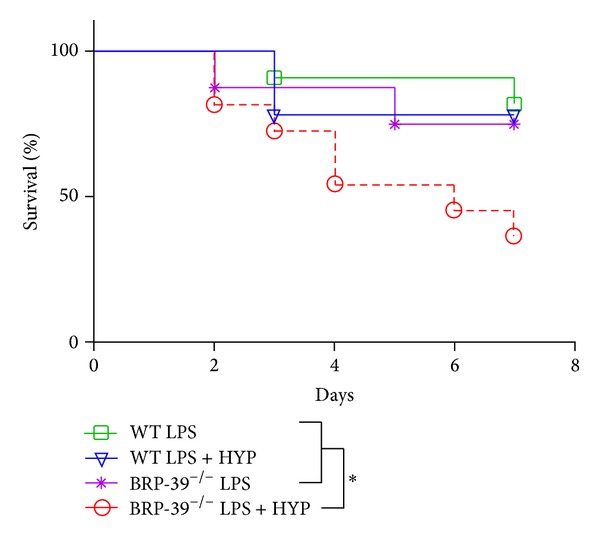
BRP-39 deletion enhances mortality in neonatal mice exposed to LPS combined with hyperoxia.**  **Newborn (NB) BRP-39^−/−^ or wild-type (WT) mice were exposed to 100% O_2_ from postnatal (PN) PN1-7 and survival was assessed. The groups were as follows: WT mice pups treated with lipopolysaccharide (LPS; *n* = 7), WT mice pups treated with LPS plus hyperoxia (HYP) (*n* = 3), BRP-39^−/−^ mice pups treated with LPS (*n* = 11), and BRP-39^−/−^ mice pups treated with LPS plus HYP (*n* = 8). LPS treatment consisted of intranasal administration on alternate days (PN2, -4, and -6) with 3 *μ*g/3 *μ*L in presence or absence of 100% oxygen. *P < 0.05.

In survival studies, WT and BRP-39^−/−^ neonatal mice underwent 100% oxygen or RA exposure for 7 days along with intranasal instillation with LPS (3 **μ**g/3 **μ**L) on alternate days (PN2, -4, and -6). Mortality was 20% at 7 days after LPS alone or with hyperoxia exposure in WT mice (Figure 6). In BRP-39^−/−^ neonatal mice, the mortality was also 20% at 7 days after LPS alone. However, it significantly increased to almost 60% in animals which received exposure of LPS combined with hyperoxia, by PN7 (Figure 6).

### 3.5. BRP-39 Modulates Oxygen-Induced Augmentation of Neonatal Lung Injury

**Figure 7:**
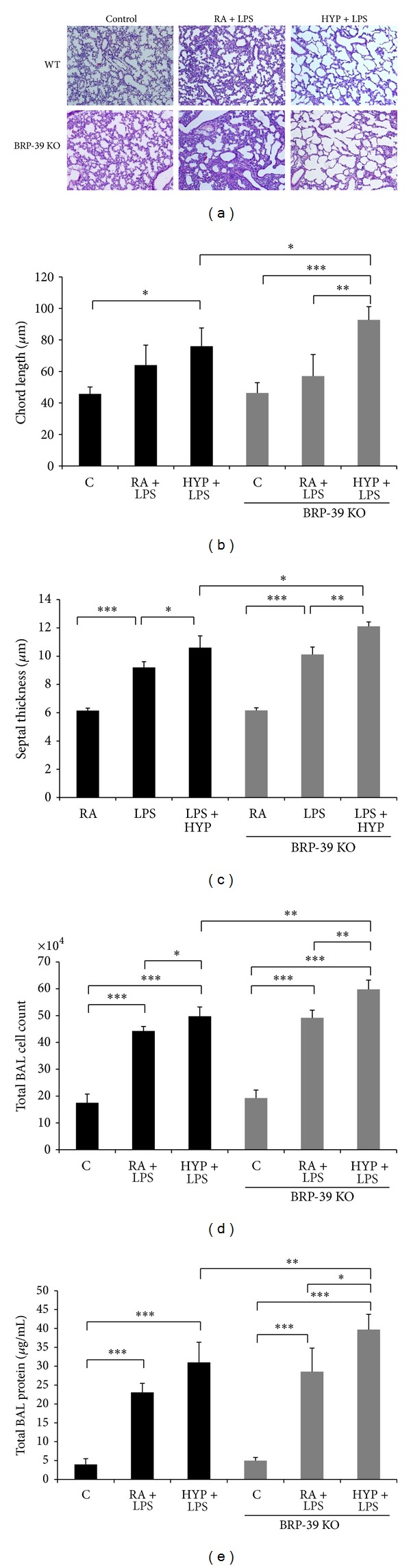
BRP-39 modulates LPS induced hyperoxia augmentation of neonatal lung injury.**  **Newborn (NB) BRP-39^−/−^ or wild-type (WT) mice were treated with LPS intranasal administration (3 **μ**g/3 **μ**L) on alternate days (postnatal or PN2, -4, -6) in presence or absence of 100% O_2_ from PN1–7. Representative photomicrographs of lung histology (H&E stain, 10x) of NB BRP-39^−/−^ or WT mice exposed to room air (RA) or hyperoxia (HYP) or LPS as noted above are shown at PN7 (a). The figures are illustrative of a minimum of 3 animals in each group. Alveolar size, as measured by chord length and septal thickness, confirmed features noted on lung histology ((b) and (c)). Each bar represents the mean ± SEM of a minimum of three animals. Bronchoalveolar lavage (BAL) total cell counts (d) and protein levels (e) of NB BRP-39^−/−^ or WT mice exposed to RA or HYP model, or given treatment as noted above, at PN7. Each bar represents the mean ± SEM of a minimum of three animals. C: control (RA); HYP: hyperoxia; BRP39 KO: BRP39 knock out or BRP-39^−/−^. *P < 0.05, **P < 0.01, and ***P < 0.001.

To begin assessing BRP-39 mediated differences in alveolar inflammation, we measured lung bronchoalveolar lavage (BAL) protein levels and inflammatory cell counts and assessed lung histology. Lung injury at PN7 was significantly increased in LPS plus oxygen-exposed BRP-39^−/−^ mice compared with similarly treated WT mice and all other treatment groups (Figure 7). In this group, we observed a significant increase in histologic damage (Figure 7(a)), chord length (Figure 7(b)), septal thickness (Figure 7(c)), BAL total cell counts (Figure 7(d)), and BAL protein (Figure 7(e)) compared with all other groups.

**Figure 8:**
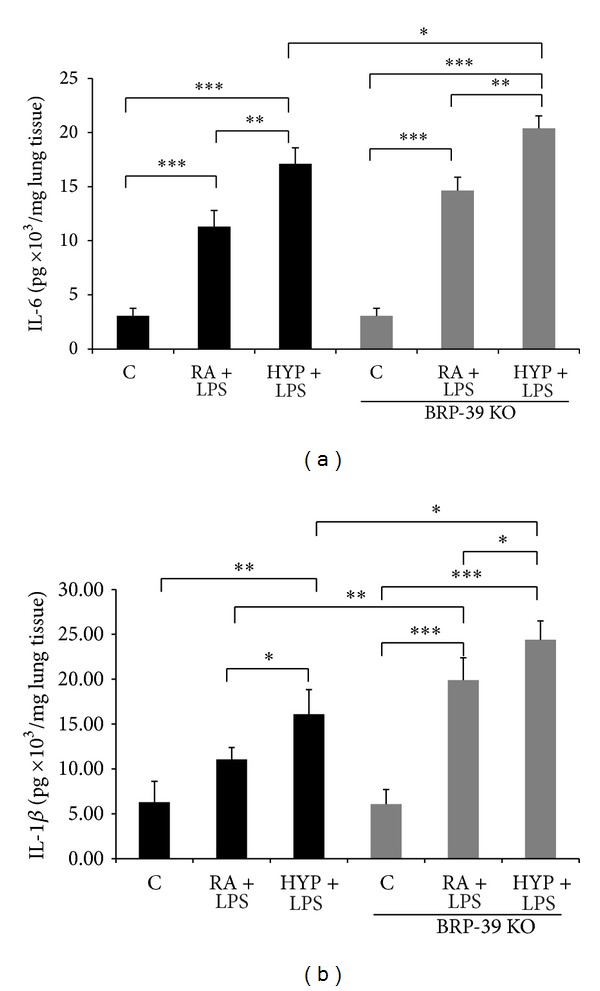
BRP-39 deletion promotes proinflammatory cytokines in neonatal mice exposed to LPS combined with hyperoxia.**  **Newborn (NB) BRP-39^−/−^ or wild-type (WT) mice were treated with LPS intranasal administration (3 **μ**g/3 **μ**L) on alternate days (postnatal or PN2, -4, -6) in presence or absence of 100% O_2_ from PN1–7. Interleukin-6**  **(IL-6) and IL-1*β* levels were measured in lung tissue homogenates of indicated treatment groups of WT and BRP-39^−/−^ mice ((a) and (b)). Each bar represents the mean ± SEM of a minimum of five animals. Results represent three independent experiments. C: control (RA); HYP: hyperoxia; BRP39 KO: BRP39 knock out or BRP-39^−/−^. *P < 0.05, **P < 0.01, and ***P < 0.001.

In BRP-39^−/−^ neonatal mice, LPS plus oxygen augmented lung injury, so we sought to determine whether M1 macrophages were responsible for this proinflammatory state. Therefore, we measured proinflammatory M1 cytokine markers IL-6 and IL-1**β** in lung homogenates. IL-6 and IL-1**β** were significantly increased in BRP-39^−/−^ neonatal mice lungs exposed to LPS plus oxygen compared with WT mice exposed to LPS plus oxygen and other experimental groups (Figures 8(a) and 8(b)).

To summarize, our data would suggest that LPS with concomitant hyperoxia exposure-induced mortality and lung injury in neonatal mice is regulated, in part, by BRP-39. The mechanism of these effects appears to be mediated, at least in part, by enhancement of the proinflammatory state, including increased M1 markers, noted in the absence of BRP-39.

## 4. Discussion

We have identified a novel role for the BRP-39 protein in the regulation of macrophage polarization *in vitro* and *in vivo*. A lack of BRP-39 in macrophages promotes a heightened responsiveness to LPS and LPS plus hyperoxia with respect to expression of genes that characterize M1 classically activated macrophages. Furthermore, *in vivo* studies utilizing a LPS and hyperoxia induced lung injury in neonatal mice reveal that BRP-39 absence in myeloid cells promotes exacerbation of disease, which is associated with an increased expression of M1 cytokines, as well as aberrant lung structure. Collectively, these findings identify BRP-39 as a modulator of macrophage activation and M1/M2 polarization, which has an impact on mortality and lung injury upon LPS and hyperoxia exposure in neonatal mice.

Macrophages are central mediators of the inflammatory response, contributing both to the initiation and the resolution of inflammation [10, 31, 32]. Activated macrophages can be M1 or M2 polarized. The importance of the macrophage inflammatory state in animal models of lung injury has been increasingly recognized [32–34]. The present studies were undertaken to first define the role of hyperoxia in M1/M2 polarization and its effect on acute lung injury. Secondly, we wanted to demonstrate the role of BRP-39 in M1/M2 macrophage polarization in postnatal inflammatory and hyperoxia induced acute lung injury conditions, both factors being responsible, at least in part, for development of the neonatal disease BPD [1, 5]. We used the LPS and LPS plus hyperoxia-exposed WT and BRP-39^−/−^ primary macrophages and lung injury models to test our hypotheses.

We showed that the hyperoxia augmented LPS induced proinflammatory M1 macrophage phenotype and attenuated IL-4 induced anti-inflammatory phenotype. M1 polarized macrophages showed higher levels of iNOS and IL-6 mRNA expression and protein concentration, which was augmented by hyperoxia exposure (Figure 1). A recently published study also showed hyperoxia augmented LPS induced inflammation in macrophages [35]. We have also recently reported that hyperoxia itself induces IFN*γ* in neonatal lungs [18, 23] and IFN*γ*-overexpressing transgenic mice lungs have a BPD-like phenotype in RA [18, 23]. We speculate that such a BPD phenotype may be secondary to enhanced M1 macrophage recruitment in lungs upon IFN*γ* stimulation. The importance of classically activated M1 macrophages in the pathogenesis of lung injury is also supported by several other reports that suggest that direct activation of these cells can augment tissue damage [34, 36, 37].

M2 polarized macrophages, characterized by various markers including Arg1 and Fizz1 and induced by TH2 cytokines IL-4 and IL-13, play an important role during alveolar development [33]. This has important potential clinical implications, not only for understanding normal developmental processes but also for addressing neonatal lung injury and inflammation [33]. The present study demonstrated that hyperoxia attenuated IL-4 induced M2 phenotype markers Arg1 and Fizz1, which suggests that hyperoxia inhibited M2 polarization in macrophages. Several studies have linked M2 macrophage activation state to tissue repair and regeneration [33, 38, 39] and a recent study identifies M2 macrophages localizing to sites of branching morphogenesis and increasing in number during the alveolarization stage, suggesting an important role during postnatal lung development [33].

Hyperoxia exposure and inflammation are the leading causes of lung injury of neonates with RDS leading to BPD [4, 5, 33, 40, 41]. In keeping with its importance, the cellular and molecular events that are involved in lung injury have been extensively investigated. The studies have highlighted a number of important events, including production of IL-1**β** and IL-6 [8, 22, 42, 43].

An interesting observation provided by our study is that BRP-39 was differentially expressed with M1 and M2 stimulants, showing a similar behavior as a M2 marker, even after exposure to hyperoxia; these data support BRP-39 being involved in macrophage function. Several previous studies have demonstrated that BRP-39 is a critical regulator of myeloid cell biology [8, 29, 44], but our novel observations introduce the role of BRP-39 in modulation of M1/M2 macrophage polarization for the first time, to the best of our knowledge.

Our initial evaluation revealed a significant decrease in BRP-39 expression in macrophages upon LPS, hyperoxia, and LPS plus hyperoxia groups exposure as compared to control groups. In accord with these findings, our group has already reported that the levels of tracheal aspirate YKL-40 (the human homolog of BRP-39) were lower in premature infants treated with hyperoxia for respiratory failure who subsequently developed BPD or died compared with those that did not experience these complications [8]. The decreased levels of BRP-39 upon LPS or/and hyperoxia exposure in macrophages further support our hypothesis that BRP-39 is essential for protection from lung injury by modulating macrophage polarization and is involved in the pathogenesis of BPD.

Macrophages in the postnatal lung displayed a M2 phenotype which is characteristic of macrophages involved in tissue remodeling functions [33]. In the present report, we noted that ablation of BRP-39 gives rise to an enhanced inflammatory M1 phenotype as compared to wild type upon exposure to LPS and hyperoxia. We believe that it is due to the hyperresponsiveness of macrophages to LPS signals in the presence of hyperoxia. *In vivo* and *in vitro* data presented in this report revealed that the ablation of BRP-39 resulted in enhanced M1 polarization as they expressed elevated iNOS, IL-6, and IL-1**β** levels.

Modulating the M1/M2 polarization status of macrophages can affect the severity of acute inflammatory conditions of the lung [38, 45]. Data presented in this report show that the lack of BRP-39 increases severity of lung injury and mortality in neonatal mice after LPS and hyperoxia exposure compared to WT mice, enhancing damage and inflammation, as evidenced by increased lung inflammatory cytokines, morphometry, and inflammatory cells. Our results are further supported by earlier studies showing that deletion of BRP-39 renders mice more susceptible to lung injury [8, 29].

These findings support the role of macrophage polarization in the development of LPS and hyperoxia plus LPS induced lung injury and suggests that BRP-39 plays an important role in the control of macrophage responsiveness and inflammation. Akt activation in macrophages has been described to determe the M1 and M2 phenotypes, specifically macrophage M2 differentiation that was associated with enhanced PI3 K/Akt signaling [46, 47]. A previous study from our group has reported that BRP-39 signaling activates Akt-ERK and p38 MAPK pathways [8]. So, it is possible that BRP-39 can affect the macrophage phenotype by modulating Akt signaling. Further studies would be required to define this relationship.

Thus, macrophage BRP-39 is a critical modifier of the oxygen-induced augmentation of inflammation and lung injury after intranasal LPS. In the absence of BRP-39, mice exposed to LPS plus oxygen exhibit more severe lung injury, and alveolar macrophages from these mice demonstrate an augmented proinflammatory M1 phenotype, known to be associated with poor outcomes. Furthermore, supplemental oxygen delivery along with LPS augments the macrophage proinflammatory state even in WT mice, at least in part, by attenuating BRP-39 after LPS and hyperoxia induction. Therefore, strategies to regulate BRP-39 expression in myeloid cells can be exploited to restrict the duration of M1 polarization and subsequent effects on inflammation and lung injury. However, additional studies are necessary to establish the (patho) physiological significance of our novel findings in the clinical setting.

## 5. Conclusion

In summary, we noted that a lack of BRP-39 in the developing lung led to both alveolar simplification and inflammatory pulmonary phenotype upon LPS administration, which was further worsened by hyperoxia exposure. These effects were associated with alterations in the macrophage polarization state from M2 to M1. We speculate that the M1/M2 polarization mediates the pulmonary effects of LPS or LPS plus hyperoxia in the developing lung. Our study has improved the understanding of the role of macrophage polarization in injury to developing lungs. Our findings have potential clinical relevance for addressing neonatal inflammatory disturbances of pulmonary development and highlight macrophage modulation as a potential intervention to cure BPD.
